# ALG3 Promotes Peritoneal Metastasis of Ovarian Cancer through Increasing Interaction of α1,3-mannosylated uPAR and ADAM8

**DOI:** 10.3390/cells11193141

**Published:** 2022-10-06

**Authors:** Xinyuan Cui, Xiaosong Pei, Hao Wang, Ping Feng, Huamin Qin, Shuai Liu, Qiu Yan, Jiwei Liu

**Affiliations:** 1 Department of Oncology, The First Affiliated Hospital of Dalian Medical University, Dalian 116011, China; 2 Liaoning Provincial Core Lab of Glycobiology and Glycoengineering, Dalian Medical University, Dalian 116044, China; 3 Department of Pathology, The Second Affiliated Hospital of Dalian Medical University, Dalian 116011, China

**Keywords:** ALG3, α1,3-mannosylation, uPAR, ADAM8, peritoneal metastasis, ovarian cancer

## Abstract

Peritoneal metastasis is the main cause of poor prognoses and high mortality in ovarian cancer patients. Abnormal protein glycosylation modification is associated with cancer malignancy. Elevated α1,3-mannosyltransferase 3 (ALG3), which catalyzes the α1,3-mannosylation of glycoproteins, has been found in some malignant tumors. However, the pathological significance of ALG3 and its regulatory mechanism in ovarian cancer metastasis is unclear. The results showed that the level of ALG3/α1,3-mannosylation was higher in human ovarian cancer tissues compared with normal ovarian tissues, as measured by Lectin chip, Western blot and Lectin blot analyses, as well as ovarian tissue microarray analysis. ALG3 was also correlated with the poor prognosis of ovarian cancer patients, according to survival analysis. The downregulation of ALG3 decreased the proliferation, stemness and peritoneal metastasis of ovarian cancer cells. The increase in urokinase plasminogen activator receptor (uPAR) α1,3-mannosylation catalyzed by ALG3 enhanced urokinase plasminogen activator (uPA)/uPAR activation and the interaction of uPAR with a disintegrin and metalloproteinase 8 (ADAM8), which promoted ovarian cancer peritoneal metastasis via the ADAM8/Ras/ERK pathway. Furthermore, decreased ALG3 suppressed ascites formation and the peritoneal metastasis of ovarian cancer cells in mice. This study highlights ALG3 as a potential diagnostic biomarker and prospective therapeutic target for ovarian cancer.

## 1. Introduction

Ovarian cancer is a highly lethal gynecological cancer [1]. Peritoneal dissemination at the late stage of diagnosis is a hallmark of ovarian cancer. Ovarian cancer cells in the primary tumor are disseminated to the peritoneum via peritoneal fluid or ascites [2]. Ovarian cancer is extremely prone to peritoneal metastasis. Peritoneal metastasis is often characterized by small nodes spread all over the peritoneal surface, resulting from rapid tumor growth and a strong adhesion capability with human peritoneal mesothelial cells (HPMCs) [3,4]. Ovarian cancer cells directly adhere to HPMCs as the initial step of metastasis. Once ovarian cancer cells attach to HPMCs, they invade the peritoneum, omental and bowel serosa, etc. [5]. Therefore, it is necessary to explore potential biomarkers and discover effective diagnostic and therapeutic targets for ovarian cancer peritoneal metastasis.

Glycosylation is an important post-translational modification of proteins. The glycobiology of cancer reveals that aberrant glycosylation is not only a feature of tumor metastasis but also a driver of the malignant phenotype [6,7]. The N-glycosylation of glycoproteins is the major type of protein glycomodification. There are three general types of N-linked glycans: high-mannose (oligomannose), hybrid and complex [8,9]. The different N-glycosylation types are catalyzed by specific glycosyltransferases. To date, six mannosyltransferases catalyzing different mannosyl linkages have been identified, including α1,2-mannosyltransferases (ALG11 and ALG9), α1,3-mannosyltransferases (ALG2 and ALG3), α1,6-mannosyltransferases (ALG12) and β1,4-mannosyltransferases (ALG1) [10,11]. Among these, ALG3 is the key enzyme catalyzing the attachment of the first mannose residue to the b branch of the dolichol-linked oligosaccharide, forming an N-glycosylation precursor. Insufficient ALG3 expression leads to the reduced biosynthesis of high-mannose and hybrid glycan epitopes [11,12]. Cancer-associated mannosylation alters cell-cell interactions and is linked to metastatic behaviors [13,14]. It was reported that the high-mannose glycan level was higher in ovarian cancer tissues and serum [15,16]. ALG3 has been reported to be overexpressed in cervical and radioresistant breast cancer cells [17,18]. However, the effect and the glycobiological mechanism of ALG3/α1,3-mannosylation in ovarian cancer metastasis are not clear.

Glycoproteins are involved in a variety of physiological and pathological processes [19]. The physiochemical characteristics and biological functions of a glycoprotein can be altered by glycosyltransferases and the corresponding glycan abnormalities, which are associated with tumor growth and malignant metastasis. The urokinase plasminogen activator receptor (uPAR), a glycoprotein linked to glycosylphosphatidylinositol (GPI) anchored on the cell surface, is highly expressed in the invasive areas of the tumor-stromal microenvironment in cancers [20,21]. The primary function of uPAR is to bind to uPA and convert plasminogen to plasmin. Subsequently, the plasmin degradation of the extracellular matrix contributes to the invasion of cancer cells. As an important pro-metastasis molecule, uPAR overexpression was found in patients with aggressive cancers, including ovarian cancer [22,23], while uPAR downregulation reduced tumor growth and metastatic potential [24]. The tumor growth and peritoneal implants of ovarian cancer cells in uPAR (−/−) mice were restrained [25]. It was reported that uPAR contains five possible N-linked glycosylation sites (N52, N162, N172, N200 and N233) [26]. However, whether N-glycosylation, especially α1,3-mannosylation by ALG3, is associated with ovarian cancer metastasis is not known. Furthermore, it is considered that the glycans of a glycoprotein mediate intermolecular recognition and interaction and initiate intracellular signaling activation to promote tumor metastasis. Being a GPI-anchored structure, uPAR needs to cooperate with adaptor molecules to transmit its intracellular signal. In this study, we hypothesized that uPAR α1,3-mannosylation modification affects the binding of uPAR to a disintegrin and metalloproteinases (ADAMs), which are transmembrane receptors identified by IP and confocal microscopy, thus activating intracellular signaling and promoting tumor metastasis.

Using Lectin chip analysis, we found that α1,3-mannosylation was significantly increased in metastatic ovarian cancer tissues. ALG3 promoted proliferation, stemness and metastasis in vitro. In addition, ALG3 facilitated metastatic potential by inducing the α1,3-mannosylation of uPAR, whereas uPAR N-glycosylation site mutation and the inhibition of α1,3-mannosylation by ALG3 downregulation suppressed ovarian cancer metastasis. Furthermore, decreased α1,3-mannosylated uPAR weakened its interaction with ADAM8 and inactivated the Ras/ERK signaling pathway. An in vivo ovarian cancer mouse model also indicated that elevated α1,3-mannosylation by ALG3 drives ovarian metastasis. This study provides new insight into the mechanism of protein glycosylation modification in ovarian cancer peritoneal metastasis.

## 2. Materials and Methods

### 2.1. Tissue Samples

Fresh ovarian cancer tissues (*n* = 3) and normal ovarian tissues (*n* = 3) from subjects aged 49−57 years were obtained from The Second Affiliated Hospital of Dalian Medical University (Dalian, China). Human normal ovarian and ovarian cancer tissue microarrays were obtained from Wellbio (Shanghai, China), which included high-grade serous ovarian cancer (*n* = 47), clear cell ovarian cancer (*n* = 9), endometrioid ovarian cancer (*n* = 14), low-grade serous ovarian cancer (*n* = 2), mucinous ovarian cancer (*n* = 9), squamous cell cancer (*n* = 1), ovarian metastasis from gastric cancer (*n* = 3), ovarian metastasis from bowel cancer (*n* = 3) and normal ovary (*n* = 8). The sample collection and all experimental protocols for the human study were in accordance with guidelines approved by the Institutional Review Boards of Dalian Medical University.

### 2.2. Data Collection

Microarray-based gene expression data (GSE18520), including ovarian tissues of ovarian cancer patients and healthy normal women, were accessed from Gene Expression Omnibus (GEO; http://www.ncbi.nlm.nih.gov/geo, accessed on 10 February 2021). The sample size of the tumor dataset was at least 10. RNA sequencing data containing 426 ovarian cancer and 88 normal samples were downloaded from Gene Expression Profiling Interactive Analysis (GEPIA; http://gepia.cancer-pku.cn/detail.php, accessed on 10 February 2021).

### 2.3. Kaplan-Meier Survival Analysis

Kaplan-Meier survival analysis used the publicly available database Kaplan-Meier plotter (http://kmplot.com/analysis, accessed on 2 March 2021). In the ovarian cancer gene dataset, a correlation analysis was performed with the following options: one probe for ALG3 (207396_s_at); patients split by the median; follow-up threshold: 60 months; survival: OS; and use of the following dataset(s) for the analysis: GSE18520.

### 2.4. Cell Culture

Human ovarian cancer A2780 and SKOV3 cells (ATCC, Manassas, VA, USA) and murine ovarian cancer ID8 cells (ATCC, Manassas, VA, USA) were cultured in RPMI 1640 (Gibco, Waltham, MA, USA), and human peritoneal mesothelial cells (HPMCs) were cultured in DMEM (Gibco, Waltham, MA, USA) supplemented with 10% fetal bovine serum (FBS; Gibco, Waltham, MA, USA) and 100 U/mL penicillin and streptomycin. Cells were grown in a humidified incubator at 37 °C in a humidified atmosphere of 5% CO_2_. When cells reached 80% confluence, they were treated with ADAM8 antibody (Santa, Dallas, TX, USA) for up to 48 h for further experimental analyses.

### 2.5. Cell Transfection

ALG3 siRNA sequences were designed and synthesized by GenePharma (Shanghai, China). ALG3 siRNA sequences were as follows: 5’-CCAGUUCUACGUCUGGUAUTT-3’ (sense); 5’-AUACCAGACGUAGAACUGGTT-3’ (antisense). TheALG3 cDNA, uPAR cDNA and uPAR MUT cDNA (N-glycosylation site mutations with N52Q, N162Q, N172Q, N200Q and N233Q replacements) were designed and synthesized by GenePharma (Shanghai, China). A2780 and SKOV3 cells were seeded onto plates. When cells reached 70% confluence, they were transiently transfected with scramble siRNA, ALG3 siRNA, vector, ALG3 cDNA, uPAR cDNA or uPAR MUT cDNA using Lipofectamine 2000 reagent (Invitrogen, Waltham, MA, USA). The samples were collected for gene and protein detection after 48 h.

### 2.6. Western Blot and Lectin Blot

Proteins extracted from cell lysates were electrophoresed in SDS-PAGE gel and transferred onto a nitrocellulose membrane. After blocking with 5% skimmed milk for 2 h, membranes were incubated with primary antibodies, as follows: ALG3 (1:1000), E-cadherin (1:1000), N-cadherin (1:1000), Snail (1:1000), Vimentin (1:1000), Ki67 (1:1000), PCNA (1:1000), Cyclin E1 (1:1000), p27 (1:1000), p21 (1:1000), Nanog (1:1000), OCT4 (1:1000), Ras (1:1000), p-ERK (1:1000), ERK (1:1000) and GAPDH (1:4000) purchased from Proteintech (Wuhan, China); ADAM8 (1:1000) and uPAR (1:1000) purchased from Santa Cruz (Santa Cruz, CA, USA); and uPA (1:1000) purchased from Abcam (Cambridge, UK) at 4 °C overnight. Then, membranes were incubated with HRP-conjugated goat anti-rabbit or anti-mouse IgG (1:2000) for 1 h. For GNA Lectin blots, the membranes were incubated with biotin-labeled Galanthus Nivalis Agglutinin (GNA, 1:2000, Vector Laboratories, Newark, CA, USA) at 4 °C overnight, followed by incubation with HRP-conjugated streptavidin (1:4000) for 45 min. An enhanced chemiluminescence (ECL) detection system (Bio-Rad, Hercules, CA, USA) was used to visualize immunoreactive bands.

### 2.7. Lectin Chip

The Lectin chip, purchased from Raybiotech (Guangzhou, China), contains 70 Lectins, which recognize and bind to different glycan structures. Normal and ovarian cancer tissues were collected and washed three times with PBS (pH = 7.4). BCA quantification was conducted with a Micro BCA Assay Kit (Thermo Scientific, Waltham, MA, USA) after tissue lysis and biotin labeling of the sample. After blocking, the sample (100 μL) was added to each well and incubated at room temperature for 1–2 h. The samples were washed 5 times, followed by incubation with Cy3-equivalent-dye-streptavidin for the detection and analysis of the fluorescent signal intensity.

### 2.8. Immunoprecipitation 

Immunoprecipitation was performed using protein A/G Immunoprecipitation (Invitrogen, USA) according to the manufacturer’s protocol. Briefly, cells were lysed in lysis buffer (50 mM Tris, 150 mM NaCl, 2 mM EDTA, protease inhibitor cocktail (Sigma, Rahway, NJ, USA) and 0.5% Triton X-100) and incubated for 1 h on ice. The cell lysate was centrifuged for 10 min at 4 °C. Protein A/G-agarose beads were incubated with primary antibodies at room temperature with mild shaking for 2 h. Next, the cell lysate (1 mg) was incubated with 5 μg of primary antibody per IP at room temperature for 2 h and washed three times. Beads were resuspended in SDS sample buffer and boiled for 10 min. For Western blots and Lectin blots, samples were resolved by SDS-PAGE and transferred to NC membranes. The membranes were incubated with biotinylated GNA Lectin (1:2000), uPA (1:1000) and ADAM8 (1:1000) at 4 °C overnight, followed by incubation with HRP-labeled streptavidin or HRP-conjugated goat anti-mouse IgG at room temperature for 1 h. An enhanced chemiluminescence (ECL) detection system (Bio-Rad, Hercules, CA, USA) was used to visualize immunoreactive bands.

### 2.9. Immunohistochemistry/Lectin Histochemistry

Ovarian tissue sections were incubated with xylol for deparaffinization and rehydration in descending concentrations of ethanol, followed by antigen retrieval in citrate buffer. A 0.3% H_2_O_2_ solution was used to remove endogenous peroxidases by incubation for 30 min, and then blocking was performed with goat serum for 45 min at room temperature. Next, the primary antibodies ALG3 (1:100) and GNA Lectin (1:300) were applied at 4 °C overnight, followed by incubation with the second antibody. The sections were visualized with diaminobenzidine (DAB) and counterstained with hematoxylin. Images were taken under an inverted microscope (Olympus, Tokyo, Japan). The yellowish-brown staining of the tissue indicated a positive result and was analyzed by Image J software.

### 2.10. Lectin ELISA

Briefly, 100 μL of the anti-uPAR (1:200) antibody diluted with PBS (1 μg/mL) was added to the wells of the plate for antibody coating. After overnight incubation at 4 °C, the plate was washed three times with 200 μL of PBST buffer. Cell lysates (100 μL, 1 mg/mL) from A2780 and SKOV3 cells were added and incubated for 2 h. After washing, biotin-labeled GNA (1:500) was added and incubated for 1 h under shaking at 37 °C. After gently washing, 50 µL of the substrate reagent for coloring was added and left for 15 min. Then, 50 µL of stop solution was added. The optical density value was read at 450 nm in an ELISA reader (Thermo Scientific, Waltham, MA, USA). The standards or samples were assayed in duplicate.

### 2.11. Hematoxylin Eosin (HE) Staining

The glass slides that held the paraffin sections were placed in staining racks. The paraffin was cleared from the samples with three changes of xylene for 2 min per change. The samples were hydrated with ethanol. The samples were stained in hematoxylin solution for 3 min and in eosin Y solution for 2 min. After dehydrating the samples, xylene was used to clear the samples. A drop of neutral gum was placed over the tissue on each slide, and a coverslip was added. The slides were viewed using a microscope.

### 2.12. Immunofluorescence and Confocal Microscopy

Immunofluorescence staining was performed after fixing the cells with 4% paraformaldehyde for 30 min. After blocking with 10% normal goat serum for 2 h, the cells were incubated with uPAR (1:100) and ADAM8 (1:100) overnight at 4 °C. After washing with PBS, the cells were incubated with TRITC-conjugated goat anti-mouse IgG (1:100, ZSGB-BIO, Beijing, China) and FITC-conjugated goat anti-rabbit IgG (1:100, ZSGB-BIO, Beijing, China) for 1 h at room temperature. Then, the slides were incubated with DAPI (4′,6-diamidino-2-phenylindole, 1:1000) for 10 min at room temperature. Images were obtained under a fluorescence microscope. The results were analyzed using a laser confocal microscope (Leica, Wetzlar, GER).

### 2.13. Transwell Assay

For the migration assay, inserts (8 µm, Costar, Rahway, NJ, USA) were used without Matrigel. For the invasion assay, the inserts were coated with 50 µL of cold-melted Matrigel (1:9 dilution, BD, Franklin Lakes, NJ, USA) at 37 °C for 2 h. A2780 cells were resuspended in 0.2 mL of serum-free RPMI 1640 medium at a concentration of 8 × 10^5^ cells. SKOV3 cells were resuspended in 0.2 mL of serum-free RPMI 1640 medium at a concentration of 5 × 10^5^ cells. Then, 800 μL of RPMI 1640 medium with 10% FBS was added to the lower chamber. After culturing at 37 °C for 19−24 h, the inserts were fixed with methanol and stained with crystal violet for 20 min. Representative images are shown.

### 2.14. Scratch Assay

After transfection with ALG3 siRNA or ALG3 cDNA, cells were scratched with a pipette tip at 80−90% confluency. Wounded cultures were incubated at 37 °C for 24 h. Subsequently, three random fields at the lesion border were observed and photographed using a microscope.

### 2.15. 5-Ethynyl-2′-deoxyuridine (EdU) Incorporation Assay

Transfected A2780 and SKOV3 cells were counted in a counting plate and seeded in a 24-well plate at 2 × 10^5^ cells per well. After the cells adhered, every other day, EdU labeling (Alexa Fluor 488) was performed with RPMI 1640 medium (containing 10% FBS) in a ratio of 1000:1, and incubation was performed in a conventional incubator for 2 h. Cells were fixed with 4% paraformaldehyde at room temperature for 30 min, and then glycine (2 mg/mL) was added to cells on a shaking bed for 5 min. Washing was performed using 100 μL of 0.1% Triton X−100 on a shaker for 10 min. Then, 1× Apollo staining solution was added and incubated on a shaker for 30 min in the dark. Finally, 250 μL of the dye solution was added to each well for nuclear staining and incubated in the dark for 30 min. Images were obtained under a fluorescence microscope.

### 2.16. Sphere Formation Assay

A2780 and SKOV3 cells were harvested and counted in the tumor sphere formation assay. Sphere-forming cells were established by suspension culture in stem-cell-conditioned medium (containing 1 mL of serum-free RPMI 1640 medium supplemented with 2% B27 (Invitrogen, Waltham, MA, USA), human recombinant fibroblast growth factor 2 (FGF−2, 20 ng/mL, Peprotech, Rocky Hill, NJ, USA), epidermal growth factor (EGF, 20 ng/mL, Peprotech, Rocky Hill, NJ, USA) and the antibiotics penicillin and streptomycin using Corning ultralow-attachment 6-well plates (Corning, NY, USA). After one week of incubation, the spheres generated were photographed, and the sphere numbers were counted under a light microscope.

### 2.17. Adhesion to Mesothelial Cells

Human peritoneal mesothelial cells (HPMCs) were grown on 96-well plates. A2780 and SKOV3 cells were prelabeled with 10 mmol/L Cell Tracker Green (CMFDA) for 1 h at 37 °C. The labeled cells were washed, and 4 × 10^3^ cells per well were added to HPMCs in 4 replicates. After incubation for the indicated times, nonadherent cells were washed. Cells were observed and photographed under an inverted microscope.

### 2.18. Molecular Docking

The Protein Data Bank (PDB) database (https://www.rcsb.org/, accessed on 18 August 2022) was used to download the “.pdb” format of the corresponding targets. The target protein structures were as follows: ADAM8 (PDB, ID: 4DD8) and uPAR (PDB, ID: 2FD6). Molecular docking was performed using the HDOCK server to analyze the interaction between uPAR and ADAM8, the amino acid sequence of which was acquired from GenBank. The binding energy ≤ −5.00 KJ/mol was selected here as the basis for screening the active compound.

### 2.19. Peritoneal Metastasis Mouse Model

Six-week-old female C57BL/6 mice were obtained from the Institute of Genome Engineered Animal Models for Human Disease (Dalian Medical University), and approval for the study was obtained from the Attitude of the Animal Care & Welfare Committee. All mice were maintained in filtered-air laminal-flow cabinets under specific pathogen-free conditions. The treatment and care of the animals were in accordance with Institutional Guidelines and the Animal Welfare Assurance Act. After 1 week of acclimation, mice received an intraperitoneal injection of 200 μL of either 5 × 10^6^ ID8 (OC group, *n* = 5), ID8-ALG3 siRNA (si-ALG3 group, *n* = 5) or ID8-GNA blockade (GNA-, *n* = 5) cells. Mice were monitored by TAUS imaging for ascites progression. When mice became unresponsive or death was imminent, exhibited respiratory difficulty or hypothermia, or showed any signs of distress, such as hunched posture, ruffled fur and reduced motility, then ascitic fluid and peritoneal specimens were collected for further investigation.

### 2.20. Statistical Analyses

All results are presented as the mean ± standard deviation (SD) from three independent experiments performed in triplicate. Statistical analyses were performed using SPSS statistical software. Survival curves were plotted using the Kaplan-Meier method, and log-rank tests were performed. The *Pearson χ*^2^ test was used to analyze the relationship between uPAR and ADAM8. Statistical significance is indicated as follows: * *p* < 0.05, ** *p* < 0.01 and *** *p* < 0.001.

## 3. Results

### 3.1. Increased α1,3-mannosylation of Glycoproteins in Ovarian Cancer Tissues

**Figure 1:**
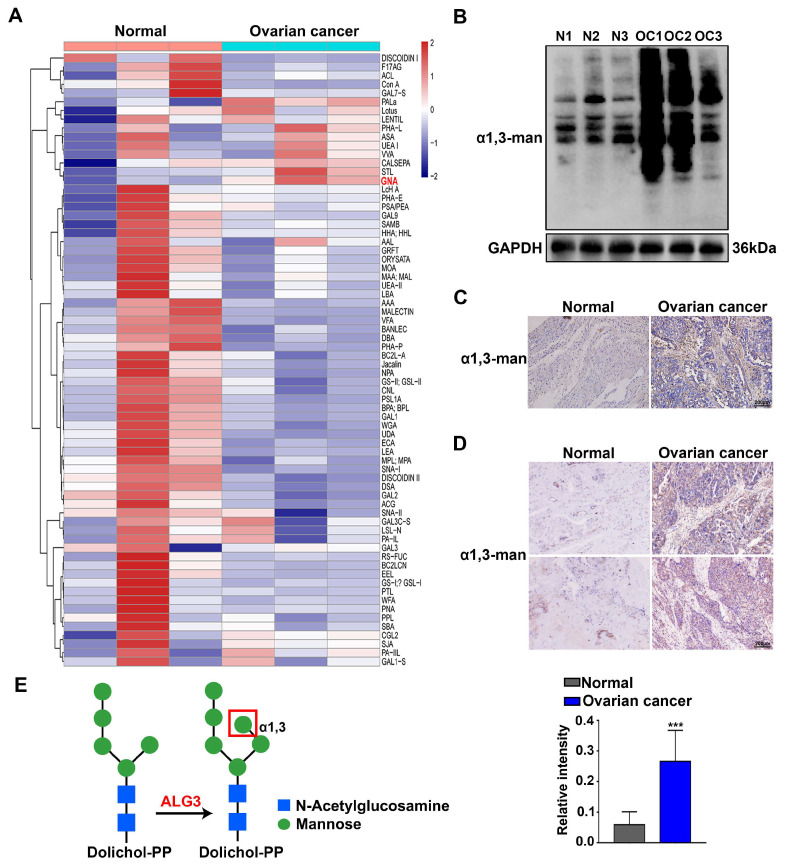
Increased α1,3-mannosylation of glycoproteins in ovarian cancer tissues. (**A**) Heatmap of Lectin clustering analysis in normal ovarian and ovarian cancer tissues (*n* = 3 pairs) measured by the Lectin chip. (**B**) Lectin blot analysis to detect α1,3-mannosylation in normal ovarian (N1–N3) and ovarian cancer tissues (OC1−OC3). (**C**,**D**) Lectin histochemical staining of α1,3-mannose glycans in normal ovarian and ovarian cancer slides and tissue microarrays. Statistical analysis of the relative intensity of normal ovarian vs. ovarian cancer tissues in ovarian cancer tissue microarrays. The bar represents 200 μm. (**E**) Diagram of α1,3-mannosylation biosynthesis catalyzed by ALG3. N: normal ovary; OC: ovarian cancer. *** *p* < 0.001.

The Lectin chip, containing 70 Lectins that recognize and bind to different glycan structures of glycoproteins, was used to obtain comprehensive information about the glycosylation variations in normal human ovarian and metastatic ovarian cancer tissues. As shown in the heatmap of Lectin clustering analysis results, metastatic ovarian cancer tissues showed a higher level of α1,3-mannose glycans bound by GNA Lectin than normal ovarian tissues (Figure 1A). To further validate the α1,3-mannosylation changes, Lectin blotting and Lectin histochemistry were performed using GNA Lectin. The ovarian cancer tissues showed a stronger intensity of GNA Lectin binding on Lectin blots (Figure 1B). The results also showed that α1,3-mannosylation was elevated in both ovarian cancer tissue slides (Figure 1C) and the ovarian cancer tissue microarray compared with that in the normal ovary (Figure 1D). Based on the fact that α1,3-mannosylation on the b branch of Man5GlcNAc2-PP-Dol is catalyzed by ALG3 (Figure 1E), we next detected ALG3 expression in normal ovarian and ovarian cancer tissues.

### 3.2. Expression Level of ALG3 Is Upregulated in Ovarian Cancer Tissues

**Figure 2:**
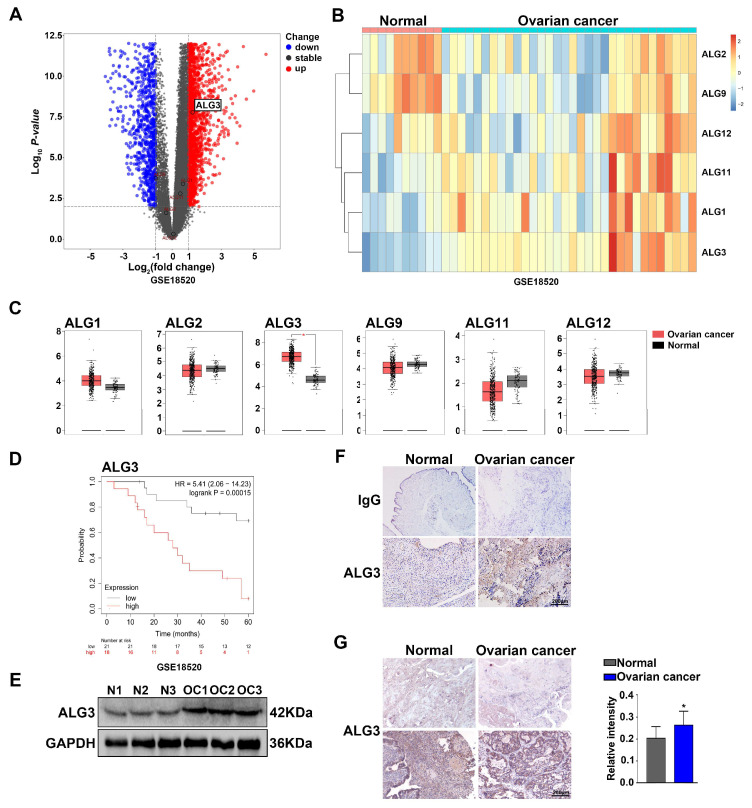
The expression level of ALG3 is upregulated in ovarian cancer tissues. (**A**,**B**) Volcano plot and heatmap of the mannosyltransferase gene (*ALGs*) in normal ovarian and ovarian cancer tissues from the GEO database (GSE18520, N = 10, OC = 32). (**C**) Differentially expressed genes (DEGs) of *ALGs* in the normal ovary and ovarian cancer from the TCGA database (N = 88, OC = 426). (**D**) Association between ALG3 mRNA expression and prognosis in ovarian cancer patients using the Kaplan-Meier plotter. (**E**) Detection of the ALG3 level in normal ovarian (N1–N3) and ovarian cancer (OC1–OC3) tissues by Western blot. (**F**,**G**) Detection of ALG3 expression in normal ovary and ovarian cancer slides and tissue microarrays by immunohistochemistry. Statistical analysis of the relative intensity of the normal ovarian tissue microarray vs. the ovarian cancer tissue microarray. The bar represents 200 μm or 50 μm. * *p* < 0.05.

To gain insight into changes in mannosyltransferases (ALGs), which have been found (ALG1, ALG2, ALG3, ALG9, ALG11 and ALG12) in ovarian cancer, we queried publicly available transcriptomic data, including Gene Expression Omnibus (GEO, GSE18520) and The Cancer Genome Atlas (TCGA) datasets. Based on GEO (GSE18520, N = 10, OC = 32), the volcano plot (Figure 2A) and heatmap (Figure 2B) showed differentially expressed genes (DEGs) of mannosyltransferases in ovarian cancer. From the TCGA database (N = 88, OC = 426) (Figure 2C), we identified that the expression of ALG3 was the highest among ALG members in ovarian cancer tissues compared with that in normal ovarian tissues. Moreover, to clarify the relationship between ALG3 expression and long-term prognosis, we used the Kaplan-Meier plotter database to analyze the association between ALG3 mRNA expression and prognosis in ovarian cancer patients. The results suggested that overall survival in patients with high expression of ALG3 in ovarian cancer was significantly shorter than those in the low-expression group (log-rank test *p* < 0.001) (Figure 2D). Furthermore, the ovarian cancer tissues showed stronger expression of ALG3 on Western blots (Figure 2E). The results also showed elevated ALG3 in both the ovarian cancer tissue slides (Figure 2F) and ovarian cancer tissue microarray compared with that in the normal ovary (Figure 2G). Collectively, these data demonstrate that ALG3 was abnormally highly expressed in ovarian cancer, and the altered expression of ALG3 would explain the changes in mannosyl glycan structures associated with the metastatic potential and poor prognosis in ovarian cancer.

### 3.3. ALG3 Promotes Cancer Stemness and Proliferation of Ovarian Cancer Cells

**Figure 3:**
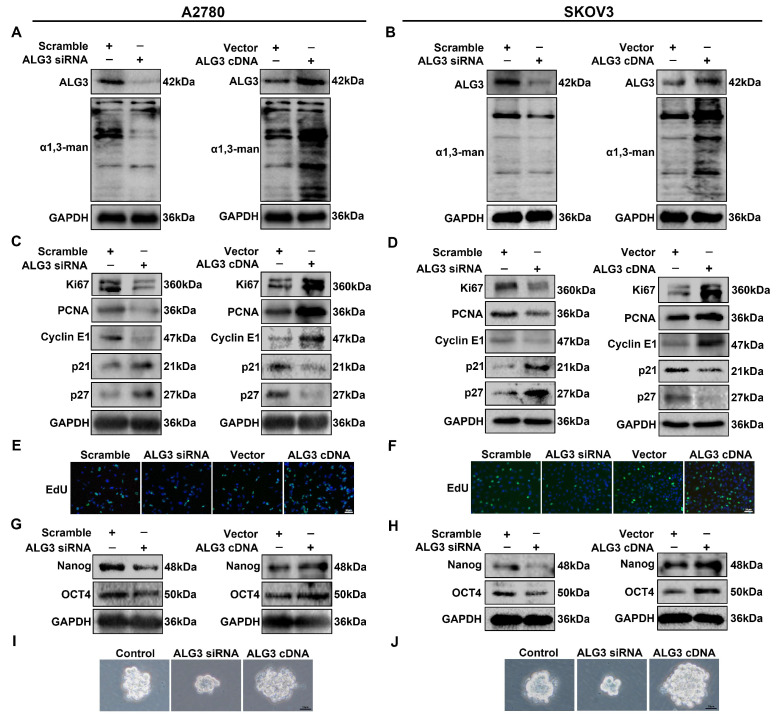
ALG3 promotes cancer stemness and proliferation in ovarian cancer cells. (**A**,**B**) Western blot analysis of ALG3 expression and Lectin blot analysis of α1,3-mannosylation in A2780 (**A**) and SKOV3 (**B**) cells after transfection with scramble siRNA, ALG3 siRNA, vector and ALG3 cDNA. (**C**,**D**) Western blot detected changes in Ki67, PCNA, cyclin E1, p21 and p27 in A2780 (**C**) and SKOV3 (**D**) cells. (**E**,**F**) EdU assay of cell proliferation ability. (**G**,**H**) Western blot analysis of Nanog and OCT4 expression. (**I**,**J**) Representative microscopy image of sphere formation assay in A2780 (**I**) and SKOV3 (**J**) cells. The bar represents 20 μm (**E**,**F**) or 10 μm (**I**,**J**).

ALG3 is the key enzyme that generates the α1,3-mannosylation epitope in the b branch of Man5GlcNAc2-PP-Dol. To explore the roles of ALG3/α1,3-mannosylation in ovarian cancer cells, A2780 and SKOV3 cells were transiently transfected with ALG3 siRNA or ALG3 cDNA. Silencing ALG3 with siRNA significantly inhibited ALG3 expression and α1,3-mannosylation, as observed by Western blot and Lectin blot, whereas transfecting ALG3 cDNA increased ALG3 expression and α1,3-mannosylation (Figure 3A,B and Figure S1(3A,B)). The Western blot results showed that the upregulation of ALG3 expression increased the intensity of proliferation-associated markers (Ki67, PCNA and Cyclin E1), and anti-proliferation markers (p21 and p27) were decreased (Figure 3C,D and Figure S1(3C,D)). We further examined the proliferative capacity of ovarian cancer cells using the EdU assay. The results showed that the knockdown of ALG3 significantly inhibited the proliferation of ovarian cells, whereas transfection with ALG3 cDNA increased the proliferative capacity (Figure 3E,F). To investigate whether ALG3 could affect ovarian cancer stem-like traits, the expression of stemness markers (Nanog and OCT4) was detected by Western blot. As shown in Figure 3G,H and Figure S1(3G,H), ALG3 overexpression increased the levels of Nanog and OCT4 in ovarian cancer cells. To verify whether ALG3 regulated the stem-like function, a sphere formation assay was implemented, and the results showed that ALG3 cDNA promoted the formation of spheres, while ALG3 siRNA decreased the formation of spheres (Figure 3I,J). The results indicate that ALG3 overexpression stimulated the proliferation and stemness of ovarian cancer cells.

### 3.4. ALG3 Enhances the Metastasis Capacity of Ovarian Cancer Cells

**Figure 4:**
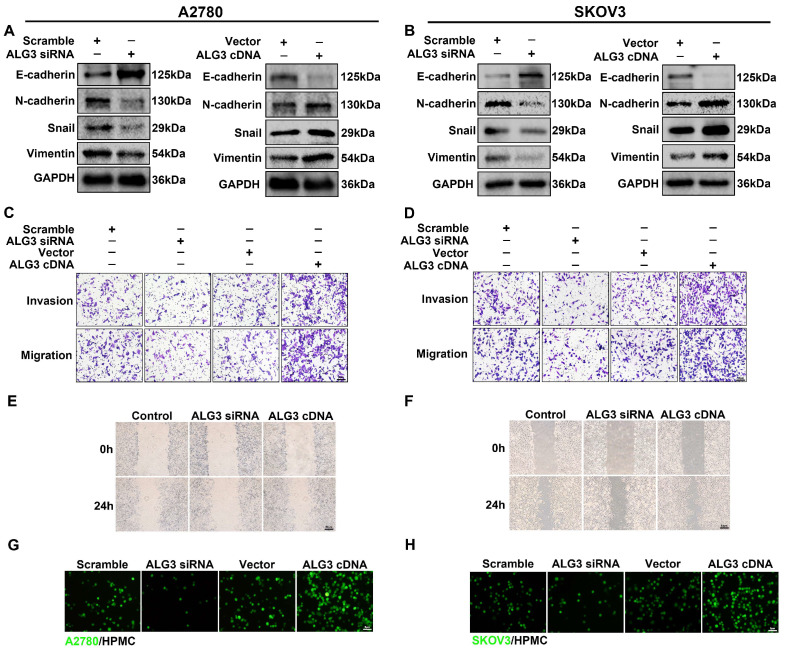
ALG3 enhances the metastasis capacity of ovarian cancer cells. (**A**,**B**) Western blot analysis of EMT markers (E-cadherin, N-cadherin, Snail and Vimentin) expressed in A2780 (**A**) and SKOV3 (**B**) cells after transfection with scramble siRNA, ALG3 siRNA, vector and ALG3 cDNA. (**C**,**D**) The migration and invasion potential detected by transwell migration/Matrigel invasion assay. (**E**,**F**) The migration ability detected by scratch assay. (**G**,**H**) Representative images of fluorescently labeled A2780 (**G**) and SKOV3 (**H**) cells adhering to HPMCs. Bars represent 5 μm (**G**,**H**), 10 μm (**C**,**D**) and 50 μm (**E**,**F**).

We investigated the role of ALG3 in ovarian cancer peritoneal metastasis by EMT, transwell migration/Matrigel invasion and scratch assays, as well as an adhesion assay. ALG3 knockdown increased E-cadherin expression, whereas it decreased N-cadherin, Snail and Vimentin expression (Figure 4A,B). The relative densitometric analysis results are shown in Supplemental Figure S1(4A,B). The transwell migration/Matrigel invasion assay showed that ALG3 siRNA reduced the number of cells crossing the insert membrane (Figure 4C,D). The scratch assay showed that ALG3 siRNA inhibited the motility capability, while the overexpression of ALG3 showed the opposite effect (Figure 4E,F). The effect of ALG3 on the ability of ovarian cancer cells to adhere to mesothelial cells was also detected. A2780 and SKOV3 cells prelabeled with the cellular fluorescent dye CMFDA were added on top of a human peritoneal mesothelial cell (HPMC) monolayer. Fluorescent density was directly proportional to adhered ovarian cancer cells and mesothelial cells. The results showed that ALG3 cDNA promoted the adhesion of ovarian cancer cells to HPMCs, and ALG3 siRNA decreased it (Figure 4G,H). These results highlight the role of ALG3 in the peritoneal metastasis of ovarian cancer.

### 3.5. uPAR α1,3-mannosylation by ALG3 Increases Its Interaction with ADAM8

**Figure 5:**
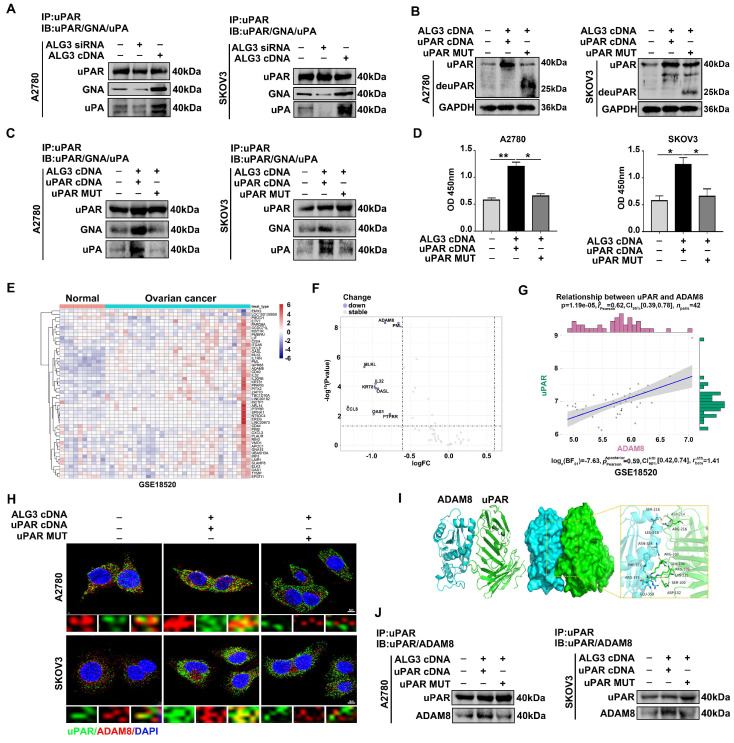
uPAR α1,3-mannosylation by ALG3 increases its interaction with ADAM8. (**A**) Immunoprecipitated uPAR after ALG3 siRNA and ALG3 cDNA transfection in A2780 and SKOV3 cells detected by GNA and uPA. (**B**) Western blot analysis of uPAR expression after uPAR cDNA and uPAR MUT cDNA transfection in A2780 and SKOV3 cells. (**C**) Anti-uPAR Immunoprecipitations from A2780 and SKOV3 cells co-transfected with ALG3 cDNA and uPAR cDNA or uPAR MUT (mutation) cDNA and blotted with biotinylated GNA and anti-uPA. (**D**) Lectin ELISA detection of glycosylated uPAR was performed in A2780 and SKOV3 cells. (**E**) Heatmap analysis of 50 genes correlated with uPAR in GSE18520. (**F**) Volcano plot of differentially expressed genes between normal ovary and ovarian cancer in GSE18520. (**G**) Correlation analysis of uPAR and ADAM8 in GSE18520. (**H**) Confocal immunofluorescence shows the location of uPAR and ADAM8. The bar represents 5 μm. (**I**) The binding mode of the complex of ADAM8 with uPAR. Green: uPAR. Bright blue: ADAM8. (**J**) Immunoprecipitation shows the binding ability of uPAR and ADAM8. * *p* < 0.05, ** *p* < 0.01.

Differential glycosylation can affect the biological functions of glycoproteins. Based on the fact that uPAR is a highly glycosylated protein, which is overexpressed in ovarian cancer, we further investigated the role of uPAR glycosylation in ovarian cancer metastasis. The results of the Immunoprecipitation (IP) of uPAR followed by GNA Lectin blotting showed reduced uPAR α1,3-mannosylation (GNA binding) in ALG3 siRNA-transfected ovarian cancer cells, whereas it increased in ALG3 cDNA-transfected ovarian cancer cells (Figure 5A and Figure S1(5A)). Meanwhile, we found decreased uPA and uPAR binding in ALG3 siRNA-transfected ovarian cancer cells compared with ALG3 cDNA transfection (Figure 5A and Figure S1(5A)). The deglycosylation of uPAR was achieved by constructing a uPAR mutation plasmid (uPAR MUT cDNA with glycosylation sites 52, 162, 172, 200 and 233 deleted). The uPAR mutation exhibited a relatively low-molecular-weight band (40 kDa–25 kDa) in Western blots (Figure 5B and Figure S1(5B)). As expected, the co-transfection of ALG3 cDNA and uPAR cDNA elevated the α1,3-mannosylation of uPAR and its binding capacity to uPA. Conversely, the co-transfection of ALG3 cDNA and uPAR MUT cDNA caused an increase in deglycosylated uPAR and decreased binding affinity (Figure 5C and Figure S1(5C)). The results of α1,3-mannosylated uPAR in the lysates of co-transfected cells showed similar changes to those before (Figure 5D).

To further clarify how α1,3-mannosylated uPAR activates downstream pathways, a correlation analysis of uPAR with target transmembrane receptors was performed. The heatmap shows the top 50 genes significantly related to uPAR identified in normal and ovarian cancer tissues (Figure 5E). The volcano plot shows that nine DEGs were upregulated in ovarian cancer, with ADAM8 the most significantly upregulated in ovarian cancer (*p* = 4.94 × 10^−9^) (Figure 5F). A positive correlation was observed between uPAR and ADAM8 (cor = 0.62, *p* = 1.19 × 10^−5^) (Figure 5G). The molecular docking data show that the binding energy of uPAR to ADAM8 was the lowest at −249.54 Kcal/mol, indicating the stable conformation between uPAR and ADAM8 (Figure 5I). The interaction was further confirmed. Confocal observations showed that uPAR and ADAM8 were co-localized in ALG3 cDNA and uPAR cDNA co-transfected cells (Figure 5H). The IP results also showed that uPAR exhibited strong interaction capability with ADAM8. However, decreased co-localization and binding of uPAR and ADAM8 in ALG3 cDNA and uPAR MUT cDNA co-transfected cells were found (Figure 5J and Figure S1(5J)). The results suggest that uPAR α1,3-mannosylation by ALG3 increases its binding with uPA and its interaction with ADAM8.

### 3.6. α1,3-mannosylated uPAR Binding to ADAM8 Stimulates Ovarian Metastasis by Activating Ras/ERK Signaling Pathway

**Figure 6:**
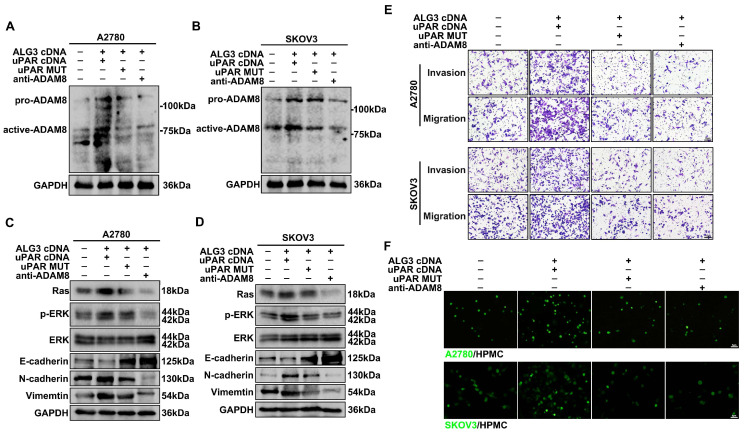
α1,3-mannosylated uPAR binding with ADAM8 stimulates ovarian metastasis by activating the Ras/ERK signaling pathway. (**A**,**B**) Western blot analysis of ADAM8 expression after ALG3 cDNA with uPAR cDNA or uPAR MUT cDNA co-transfection and antibody blockade of ADAM8 in A2780 (**A**) and SKOV3 (**B**) cells. (**C**,**D**) Western blot analysis of Ras, p-ERK and ERK expression, as well as EMT markers (E-cadherin, N-cadherin and Vimentin) expression. (**E**) The migration and invasion potential detected by transwell migration/Matrigel invasion assay. (**F**) Representative images of fluorescently labeled A2780 and SKOV3 cells adhering to HPMCs. Bars represent 5 μm (**F**) and 10 μm (**E**).

We next detected whether ADAM8 activation is regulated by the uPAR glycosylation status. ADAM8 is synthesized as an inactive proform (120 kDa) and autocatalytically yields an active form (90 kDa) after binding to specific molecules [27]. The results showed that the active form ADAM8 was increased in ALG3 cDNA and uPAR cDNA co-transfected cells but decreased in ALG3 cDNA and uPAR MUT cDNA co-transfected cells. Antibody blockade of ADAM8 (anti-ADAM8) prevented the active form of ADAM8 from forming (Figure 6A,B and Figure S1(6A,B)). We further detected the signaling cascade underlying α1,3-mannosylated uPAR binding to ADAM8 in ovarian metastasis. The results showed that the expression of downstream signaling molecules (Ras and p-ERK) of the Ras/ERK signaling pathway was increased in the uPAR cDNA and ALG3 cDNA co-transfected group, whereas the levels of Ras and p-ERK were significantly attenuated in both the uPAR MUT cDNA and anti-ADAM8 groups (Figure 6C,D and Figure S1(6C,D)). As shown in Western blots, uPAR cDNA promoted the expression of EMT markers, whereas uPAR MUT cDNA and anti-ADAM8 inhibited their expression (Figure 6C,D and Figure S1(6C,D)). The transwell assay showed that co-transfection with ALG3 cDNA and uPAR cDNA promoted the migration and invasion ability of ovarian cancer cells (Figure 6E). In the peritoneal adhesion assay, we also proved that uPAR cDNA promoted ovarian cancer adherence to HPMCs, but uPAR MUT cDNA and anti-ADAM8 decreased peritoneal metastasis (Figure 6F). Reduced α1,3-mannosylated uPAR counteracted the pro-invasive effects of uPAR overexpression. These findings reveal that uPAR α1,3-mannosylation controls the combination of uPAR and ADAM8 and promotes ovarian cancer peritoneal metastasis by activating the Ras/ERK signaling pathway.

### 3.7. Decreased α1,3-mannosylation Inhibits Peritoneal Metastasis of Ovarian Cancer In Vivo

**Figure 7:**
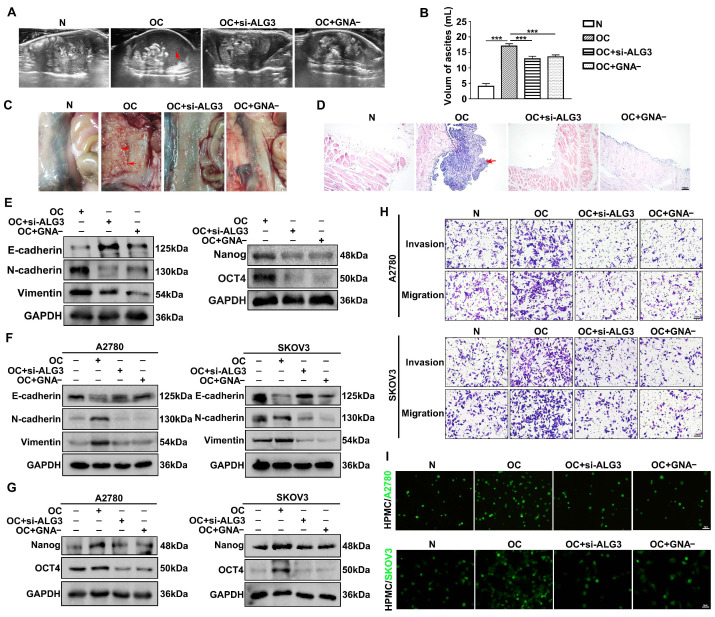
Downregulation of α1,3-mannosylation inhibits tumor peritoneal metastasis in a mouse model. (**A**) Ultrasound image to detect the amount of ascites (red arrow) in the peritoneal cavity of different groups (OC, si-ALG3 and GNA-). (**B**) Statistical analysis of the volume of ascites among the groups before mice were sacrificed. (**C**) Representative images of the cancer metastasis nodes (red arrows) in the peritoneum. (**D**) The HE-stained peritoneum was dissected longitudinally. The red arrow indicates metastatic tumor nodes. (**E**) Western blot analysis of EMT and stemness marker expression in peritoneal cancer tissues. (**F**,**G**) Western blots showing the expression of EMT and stemness markers in A2780 and SKOV3 cells treated with ascites from the mouse peritoneal cavity. (**H**) The migration and invasion potential detected by transwell migration/Matrigel invasion assay. (**I**) Representative images of fluorescently labeled A2780 and SKOV3 cells adhering to HPMCs. Bars represent 5 μm (**I**), 10 μm (**H**) and 100 μm (**D**). *** *p* < 0.001.

A peritoneal metastasis mouse model was established using ID8 murine cancer cells. The results showed that a mouse injected with cancer cells generated ascites in less than 40 days. The ascites volume was detected via transabdominal ultrasound (TAUS). Longitudinal imaging demonstrated that the amount of ascites was high in the mouse ovarian cancer group (OC) compared with that in the ALG3 downregulation group (si-ALG3) and GNA blockade group (GNA-) (Figure 7A). The OC group showed a larger volume of ascites compared with the si-ALG3 and GNA- groups (Figure 7B). The histological observation of the dissected peritoneum showed metastatic cancer nodes (Figure 7C). Specimens of the peritoneum were also investigated by HE staining (Figure 7D). The EMT markers (E-cadherin, N-cadherin and Vimentin) and stemness markers (Nanog and OCT4) were analyzed from metastatic ovarian cancer tissues. As shown in Figure 7E and Figure S1(7E), the OC group promoted the expression of EMT and stemness markers. The ascites tumor microenvironment contributes to cancer progression. As expected, Western blots showed that ascites collected from the OC group stimulated EMT (Figure 7F and Figure S1(7F)) and stemness (Figure 7G and Figure S1(7G)) by regulating the expression of the markers in A2780 and SKOV3 cells. Using the transwell migration/Matrigel invasion assay and adhesion assay, we found that ascites collected from the OC group stimulated the peritoneal metastasis of ovarian cancer (Figure 7H,I). However, the ascites of the si-ALG3 and GNA- groups significantly reduced peritoneal metastasis. Taken together, our data demonstrate that ALG3/α1,3-mannosylation is necessary for ovarian cancer cells to maintain their metastatic malignancy in the mouse abdominal cavity.

## 4. Discussion

**Figure 8:**
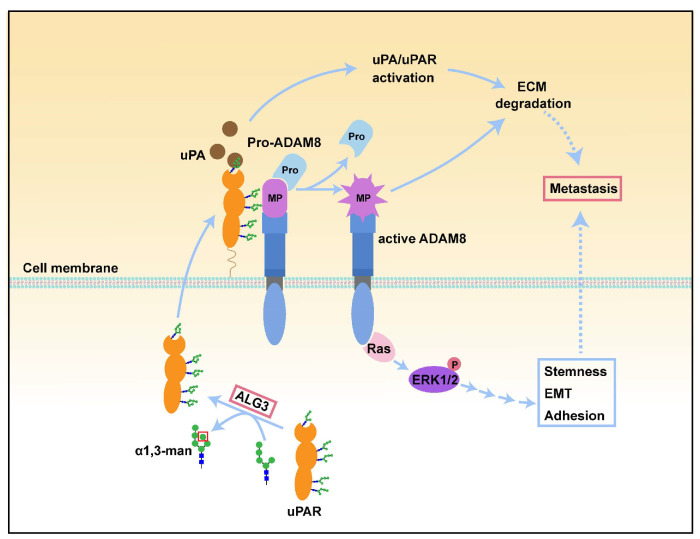
Schematic graph elucidating the underlying mechanisms through which uPAR α1,3-mannosylation catalyzed by ALG3 enhances uPA/uPAR activation and uPAR with ADAM8 interaction, which promotes ovarian cancer metastasis via the ADAM8/Ras/ERK pathway.

Aberrant glycosylation may cause tumorigenesis and malignant phenotypes, such as cell adhesion, motility, invasion and immune evasion [28,29,30]. Whether protein mannosylation modification is associated with ovarian cancer metastasis is not known. In the present study, we observed that the levels of α1,3-mannosylation and ALG3 were significantly upregulated in ovarian cancer tissues compared with normal ovarian tissues, which was closely related to metastatic potential and the poor prognosis of the patients. The in vitro and in vivo results also revealed that decreased α1,3-mannosylation by ALG3 suppressed ovarian cancer stemness and peritoneal metastasis. In addition, ALG3 was found to increase the α1,3-mannosylation of uPAR and enhance its interaction with and activation of ADAM8, thus promoting ovarian cancer metastasis (Figure 8).

Elevated mannosylation is associated with cancer metastasis capability. For example, high-mannose glycans were elevated in the human breast cancer cells and tissues, as determined by PGC-ESI-MS/MS, and were used as a potential diagnostic marker and therapeutic target in metastatic breast cancer [31]. The elevation of high-mannose glycans by inhibiting α-mannosidase I facilitate the metastatic potential of cholangiocarcinoma cells [13]. Analysis using nano-LC on porous graphitized carbon and negative-ion ESI-MS revealed increased levels of high-mannose glycans in ovarian cancer cell lines (SKOV3, IGROV1, A2780 and OVCAR3) compared to normal ovarian cell lines (HOSE6.3 and HOSE17.1) [32,33]. Here, we found that α1,3-mannose glycans of the proteins were significantly elevated in human ovarian cancer tissues compared with those in normal ovarian tissues by using the Lectin chip assay and further confirmed this result by Lectin blotting and Lectin histochemistry (Figure 1). X. Sun et al. reported that inducing the mannosylation of TGF-β receptor II (TGFBR2) by ALG3 promoted radioresistance and cancer stemness in breast cancer, whereas tunicamycin and LY2109761 abrogated the stimulatory effect of ALG3 overexpression [18]. The reduced O-mannosylation of E-cadherin in human gastric cancer cells inhibited its metastatic functions [34]. Using a peritoneal metastatic mouse model, we proved that inhibiting the level of α1,3-mannosylation reduced the formation of ascites and peritoneal metastasis (Figure 7). The study reveals that the α1,3-mannosylation of glycoproteins enhances the malignant progression of ovarian cancer.

The differential expression of glycosyltransferases has been reported as an ovarian cancer biomarker. Sialyltransferase ST3Gal1 increases α2,3-sialylation in serous ovarian cancer, promotes EMT and confers paclitaxel resistance [35]. α3/4-Fucosyltransferases (FUT3, FUT4 and FUT9) are active in ovarian cancer development [36]. Based on GEO and TCGA data searches, we found that ALG3 was upregulated in metastatic ovarian cancer and enhanced the α1,3-mannosylation of glycoproteins (Figure 2A−C). Lectin blotting and histochemistry further proved the increase in ALG3 in metastatic ovarian cancer tissues (Figure 2E−G). Moreover, ALG3 expression was linked to a poor prognosis (Figure 2D). P. Shao et al. found that ALG3 was highly expressed in esophageal squamous cell cancer, which increased proliferation and aggressive behaviors [37]. Y.W. Choi et al. reported that ALG3 is highly expressed in lymph node metastasis and also promotes the proliferative capacity of cervical cancer cells [17]. In contrast, S.B. Ke et al. reported that ALG3 knockdown inhibited EMT in non-small lung cancer cells [38]. However, the role of ALG3 in ovarian cancer progression and the underlying molecular mechanism is unknown. Our results showed that ALG3 overexpression promoted stemness, proliferation and adhesion to peritoneal mesothelial cells in ovarian cancer cells. Silencing ALG3 reduced the peritoneal metastasis of ovarian cancer. Our findings indicate that ALG3 can serve as a potential biomarker in metastatic ovarian cancer from the perspective of glycobiology.

Increased uPAR degrades the extracellular matrix to promote cancer metastasis by activating uPA/uPAR. In ovarian cancer, uPAR is abundantly expressed [22]. The glycosylation status also affects its pro-metastasis functions in cancer. Magnussen SN et al. found that the tumor microenvironment regulated uPAR expression and its glycosylation, which enhanced the migration and invasion of human oral squamous cell carcinoma [39]. Therefore, we hypothesized that uPAR glycosylation facilitates its pro-tumor effects in ovarian cancer. In the current study, we identified that uPAR is an α1,3-mannosylated glycoprotein, and the elevated α1,3-mannosylation of uPAR by ALG3 increased its binding potential with uPA. In contrast, the increase in deglycosylated uPAR by glycosylation site mutation (uPAR MUT cDNA) decreased the binding affinity (Figure 5A). uPAR is a GPI-anchored glycoprotein that requires interaction with transmembrane adapter proteins to activate intracellular signaling [40]. It was reported that β1 integrin is the co-receptor that mediates uPA/uPAR action in neurorepair [41]. EGFR acts as a transducer of the signal from uPAR to ERK in COS-7 cells [42]. In this study, we identified a new transmembrane adaptor, ADAM8, which can strongly interact with uPAR, through bioinformatics, IP and confocal analyses (Figure 5E−J). The active form of ADAM8 cleaved important ECM components and contributed to the invasiveness of cancer cells [43]. We found that after the binding of uPAR and ADAM8, the active form of ADAM8 (90 kDa) was produced, which promoted ovarian cancer metastasis. It was found that ADAM8 promoted the migration and invasion of triple-negative breast cancer cells by activating the ERK signaling cascade [44]. Our study demonstrated that reducing uPAR α1,3-mannosylation by uPAR MUT cDNA inhibited the binding of uPAR to ADAM8, which reduced active ADAM8 and thus the downstream Ras/ERK signaling pathway mediating ovarian cancer metastasis (Figure 6). This study suggests that elevated uPAR α1,3-mannosylation modification catalyzed by ALG3 increases the peritoneal metastasis ability by activating the ADAM8/Ras/ERK signaling pathway.

Collectively, our results revealed that elevated α1,3-mannosylation and ALG3 were related to metastatic ovarian cancer and a poor prognosis and promoted the proliferation, stemness and peritoneal metastasis of ovarian cancer cells. The increased α1,3-mannosylation of uPAR catalyzed by ALG3 facilitated uPAR and ADAM8 interaction. ALG3/α1,3-mannosylation may serve as a novel biomarker for metastatic ovarian cancer. Exploring the mechanism of ALG3/α1,3-mannosylation would provide new strategies for the treatment and diagnosis of peritoneal metastatic ovarian cancer from the aspect of glycobiology.

## Data Availability

Not applicable.

## Supplementary Materials

The following supporting information can be downloaded at: https://www.mdpi.com/article/10.3390/cells11193141/s1. Figure S1: Relative densitometric analysis of Western blot and Lectin blot.

## Abbreviations

N: normal ovary; OC: ovarian cancer; ALG3: α1,3-mannosyltransferase 3; uPAR: urokinase plasminogen activator receptor; uPA: urokinase plasminogen activator; ADAM8: a disintegrin and metalloproteinase 8; HPMCs: human peritoneal mesothelial cells; GPI: glycosylphosphatidylinositol; GNA: Galanthus Nivalis Agglutinin; EdU: 5-ethynyl-2′-deoxyuridine; EMT: epithelial-mesenchymal transition; CMFDA: 5-chloromethylfluorescein diacetate; IP: Immunoprecipitation; TAUS: transabdominal ultrasound.
