# Supplementary material for: ALG3 Promotes Peritoneal Metastasis of Ovarian Cancer through Increasing Interaction of α1,3-mannosylated uPAR and ADAM8
Source: Cells. 2022 Oct 6;11(19):3141. doi: 10.3390/cells11193141 (PMC9563302; doi:10.3390/cells11193141)
Supplement: Supplementary file 1 [file cells-11-03141-s001.zip › cells-1898201-supplementary.pdf]

Fig. S1 Relative densitometric analysis of Western blot and Lectin blot

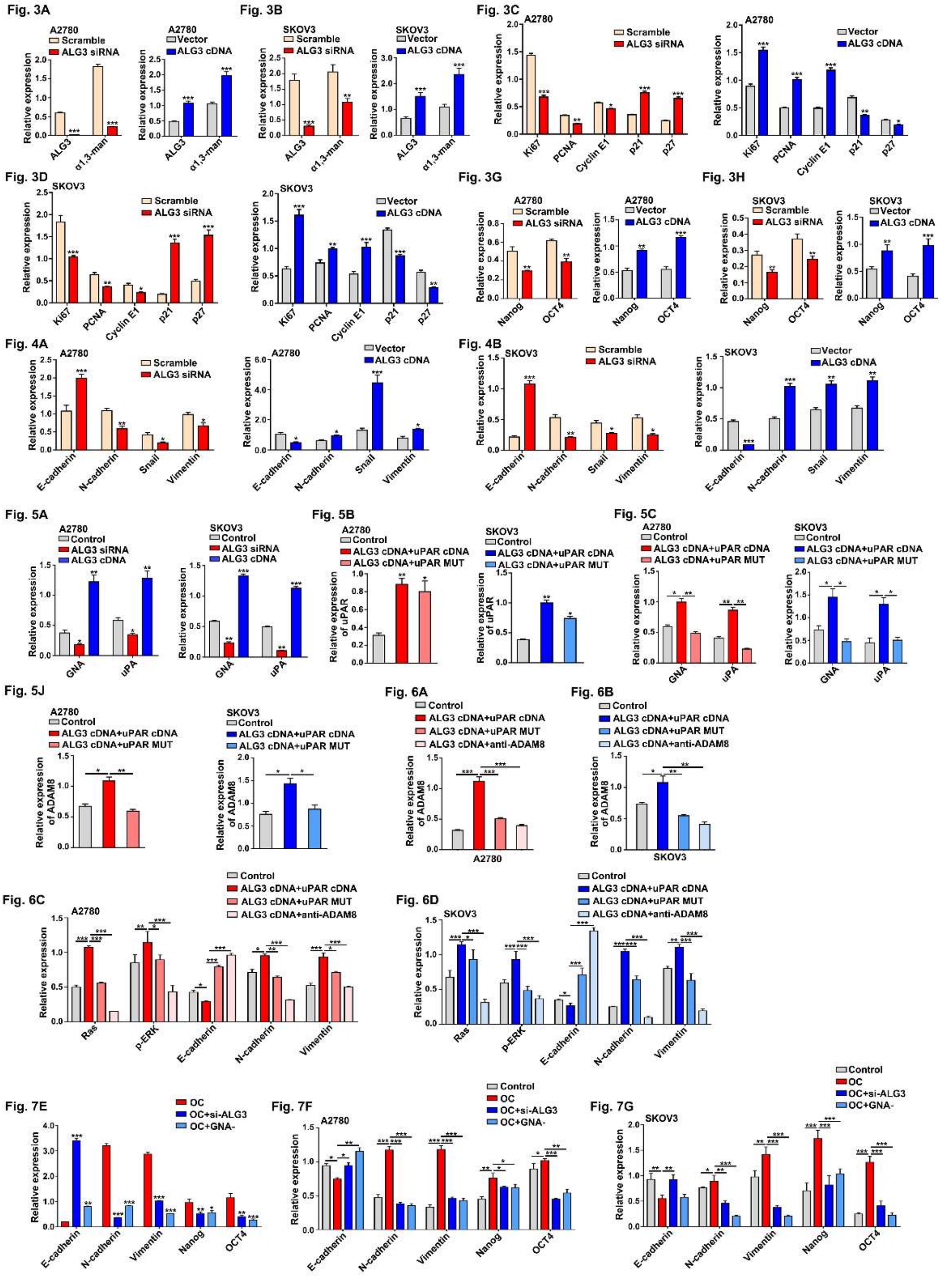

Figure S1. Relative densitometric analysis of Western blot and Lectin blot. (Fig. 3A, B) Analysis of ALG3 and α1,3-mannosylation (α1,3-man) in scramble vs ALG3 siRNA and vector vs ALG3 cDNA transfected A2780 cells **(Fig. 3A)** and SKOV3 cells **(Fig. 3B)**. **(Fig. 3C, D)** Analysis of Ki67, PCNA, CyclinE1, p21 and p27 levels in scramble vs ALG3 siRNA, and vector vs ALG3 cDNA transfected A2780 cells **(Fig. 3C)** and SKOV3 cells **(Fig. 3D). (Fig. 3G, H)** Analysis of Nanog and OCT4 levels in scramble vs ALG3 siRNA, and vector vs ALG3 cDNA transfected A2780 cells **(Fig. 3G)** and SKOV3 cells **(Fig. 3H).**

(Fig. 4A, B) Analysis of E-cadherin, N-cadherin, Snail and Vimentin levels in scramble vs ALG3 siRNA, and vector vs ALG3 cDNA transfected A2780 cells **(Fig. 4A)** and SKOV3 cells **(Fig. 4B). (Fig. 5A)** Analysis of GNA and uPA levels in control vs ALG3 siRNA or ALG3 cDNA transfected A2780 and SKOV3 cells by Immunoprecipitation (IP). **(Fig. 5B)** Analysis of uPAR level in control vs ALG3 cDNA + uPAR cDNA, and ALG3 cDNA + uPAR cDNA vs ALG3 cDNA+uPAR MUT cDNA in A2780 and SKOV3 cells. **(Fig. 5C, J)** Analysis of GNA, uPA and ADAM8 levels in control vs ALG3 cDNA + uPAR cDNA, and ALG3 cDNA + uPAR cDNA vs ALG3 cDNA + uPAR MUT cDNA in A2780 and SKOV3 cells by IP. **(Fig. 6A-D)** Analysis of ADAM8, Ras, p-ERK, E-cadherin, N-cadherin and Vimentin levels in control vs ALG3 cDNA + uPAR cDNA, ALG3 cDNA + uPAR cDNA vs ALG3 cDNA + uPAR MUT cDNA and ALG3 cDNA + uPAR cDNA vs ALG3 cDNA + anti-ADAM8 in A2780 and SKOV3 cells. **(Fig. 7E)** Analysis of E-cadherin, N-cadherin, Vimentin, Nanog and OCT4 levels in peritoneal cancer tissues (OC vs si-ALG3 and OC vs GNA-). **(Fig. 7F, G)** Analysis of E-cadherin, N-cadherin, Vimentin, Nanog and OCT4 levels in different ascites treated A2780 cells **(Fig. 7F)** and SKOV3 cells **(Fig. 7G)**. OC: ovarian cancer group, GNA-: GNA Lectin blocking group. Data were presented as mean ± SEM *$p < 0.05$, **$p < 0.01$, ***$p < 0.001$.
